# Examination of Cholesterol oxidase attachment to magnetic nanoparticles

**DOI:** 10.1186/1477-3155-3-1

**Published:** 2005-01-20

**Authors:** Gilles K Kouassi, Joseph Irudayaraj, Gregory McCarty

**Affiliations:** 1 Department of Agricultural and Biological Engineering, 249 Agricultural Engineering Building, The Pennsylvania State University, University Park, PA 16802, USA; 2 Department of Engineering Sciences and Mechanics, The Pennsylvania State University, University Park, PA 16802, USA

## Abstract

Magnetic nanoparticles (Fe_3_O_4_) were synthesized by thermal co-precipitation of ferric and ferrous chlorides. The sizes and structure of the particles were characterized using transmission electron microscopy (TEM). The size of the particles was in the range between 9.7 and 56.4 nm. Cholesterol oxidase (CHO) was successfully bound to the particles via carbodiimide activation. FTIR spectroscopy was used to confirm the binding of CHO to the particles. The binding efficiency was between 98 and 100% irrespective of the amount of particles used. Kinetic studies of the free and bound CHO revealed that the stability and activity of the enzyme were significantly improved upon binding to the nanoparticles. Furthermore, the bound enzyme exhibited a better tolerance to pH, temperature and substrate concentration. The activation energy for free and bound CHO was 13.6 and 9.3 kJ/mol, respectively. This indicated that the energy barrier of CHO activity was reduced upon binding onto Fe_3_O_4 _nanoparticles. The improvements observed in activity, stability, and functionality of CHO resulted from structural and conformational changes of the bound enzyme. The study indicates that the stability and activity of CHO could be enhanced via attachment to magnetic nanoparticles and subsequently will contribute to better uses of this enzyme in various biological and clinical applications.

## Background

Magnetic materials have been used with grain sizes down to the nanoscale for longer than any other type of material [1]. This is attributable to a number of factors including a large surface area to volume ratio and the possibility of immobilizing a biological entity of interest [2]. In the last decade increased investigations and development were observed in the field of nanosized magnetic particles [2]. Here the term nanoparticles is used to designate particulate systems that are less than 1μm, and effectively below 500 nm [2].

Due to their magnetic character, magnetite (Fe_3_O_4_) nanoparticles can be attracted by a magnetic field and are easily separable in solution. Similarly, substances to which they have been attached can be separated from a reaction medium, or directed by an external magnetic field to site specific drug delivery targets [2]. Magnetic nanoparticles have been widely used in the immobilization of many bioactive substances such as proteins, peptides, enzymes [3-6], and antibodies [7]. Magnetite is one of the most commonly used magnetic materials because it has a strong magnetic property and low toxicity [4].

The binding of magnetic particles to bioactive substances involves a number of interactions including the interactions between organic ligand, and the interactions between the amino acid side chains of proteins and the metals centers. Such bindings pave the way for the coupling of biomolecular entities of enhanced stability. Recently reported work in the area of enzyme immobilization has described the catalytic activity of yeast alcohol dehydrogenase [3] and lipase [4] directly bound to magnetite nanoparticles, via carbodimiide activation without the use of a ligand. This binding method offers tremendous scope because of its simplicity and high efficiency.

Cholesterol oxidase is a flavin-enzyme (with a FAD prosphetic group) that produces hydrogen peroxide according to the reaction 1.

*Cholesterol *+ *O*_2 _→ 4 - *Cholesten *- 3 - *one *+ *H*_2_*O*_2_   (1)

The structure of cholesterol oxidase reveals deeply buried active sites occupied by water molecules in the absence of its substrate steroids [8]. Cholesterol oxidase is industrially and commercially important for application in bioconversions for clinical determination of total or free serum cholesterol [9-12] and in agriculture [13]. Its activity can be determined by following the appearance of the conjugated ketones, the formation of hydrogen peroxide in a coupled test with peroxidase, or by measuring the oxygen consumption polarographically [13]. Several studies on its kinetic properties have appeared [13-15]. More recently, Cholesterol biosensor based on entrapment of cholesterol oxidase in a silicic sol-gel matrix at a Prussian Blue modified electrode has been developed [15]. However, this method of enzyme immobilization raises concerns on reduced surface area for enzyme binding and pore-diffusion resistance [2]. Immobilization of enzymes onto inorganic material surfaces is of vital importance in enzymatic reactions, especially in biosensor applications. Information on the activity and availability of cholesterol oxidase bound to Fe_3_O_4 _magnetic nanoparticles will contribute to the basic understanding of its activity and function.

The present study proposes to investigate the direct binding of cholesterol oxidase to Fe_3_O_4 _magnetic nanoparticles. The sizes and structure of the nanoparticles were characterized using TEM and FTIR spectroscopy. The stability, activity, and kinetic behavior of bound cholesterol were also examined.

## Results and discussions

### Particle size and structure

**Figure 1:**
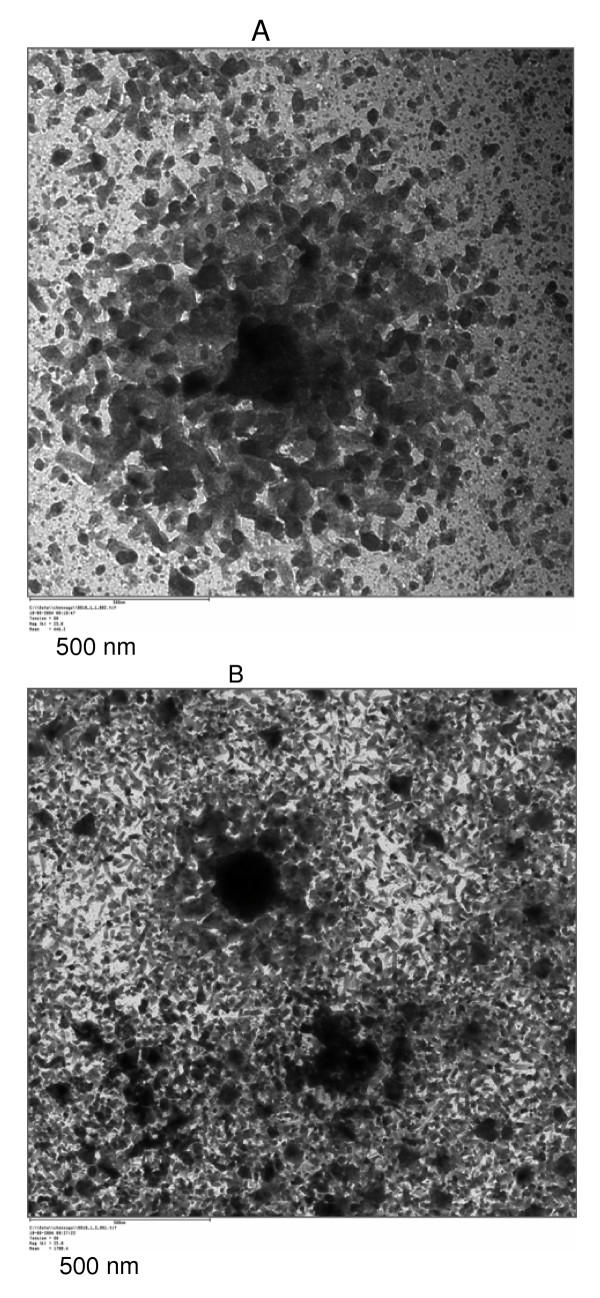
Transmssion Electron micrographs of Fe_3_O_4 _magnetic nanoparticles (a) and Fe_3_O_4_-CHO (b).

**Figure 2:**
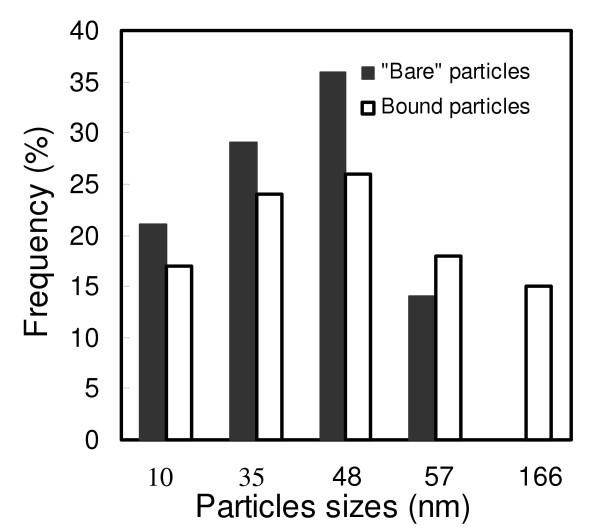
Distribution of the particle sizes on the electron micrographs. The values denote the averages of duplicate measurements.

TEM micrographs of "bare" magnetic nanoparticles and CHO-functionalized magnetic nanoparticles are shown in Figure 1a and 1b. The "bare" particles were very fine with a diameter ranging from 9.7 to 56.4 nm. The size of the particles after binding to CHO was globally the same as the "bare" particles. Figure 2 shows the size distribution of the particles. However, some spots of agglomerated particles were visible as seen in figure 1b. These agglomerates cause an increase in maximum particle size. The overall sizes of the particles after binding to magnetic nanoparticles were between 9.7 and 166 nm suggesting a perceptible agglomeration in association with the binding process. A possible explanation is that the binding of magnetic nanoparticles was not only a monomolecular process but may involve the binding of several CHO molecules on a single Fe_3_O_4 _particle. It could also be envisaged that CHO molecules formed aggregates to bind several magnetic nanoparticles. Another possible factor in the agglomeration process is the centrifugation process involved in the separation of the supernatant from the Fe_3_O_4_-CHO. It is obvious that the centrifugation tend to bring particles together as a compact material. The effect of agglomeration at this stage can be reduced by separating the Fe_3_O_4_-CHO by an external magnetic field. Since the particles are released after removal of the magnetic field, they may fall separately apart from each other, and are less likely to agglomerate.

### Binding efficiency

The unbound enzyme was determined by assaying the protein content in the supernatant. It was found that the percentage of cholesterol oxidase bound was between 98 and 100%, irrespective of the amount of particles. The amounts of Fe_3_O_4 _nanoparticles used were 14.4, 17.2 and 20 mg/mL, corresponding to CHO/Fe_3_O_4 _weight ratios of 0.01, 0.08 and 0.007, respectively. These results show that in all the binding operations, there were sufficiently available amount of particles to bind the enzymes till complete saturation. In a previous study [4], it was found that increasing the amount of Fe_3_O_4 _nanoparticles, that is reducing the weight ratio of CHO to Fe_3_O_4 _below 0.033 caused an increase in lipase binding up to 100%. This was not observed in this study, possibly because of the difference in the binding mechanism, due to differences in the structure of the enzyme. However, the percentage of bound CHO (98–100%) shows that the binding process was successful.

### Binding confirmation

**Figure 3:**
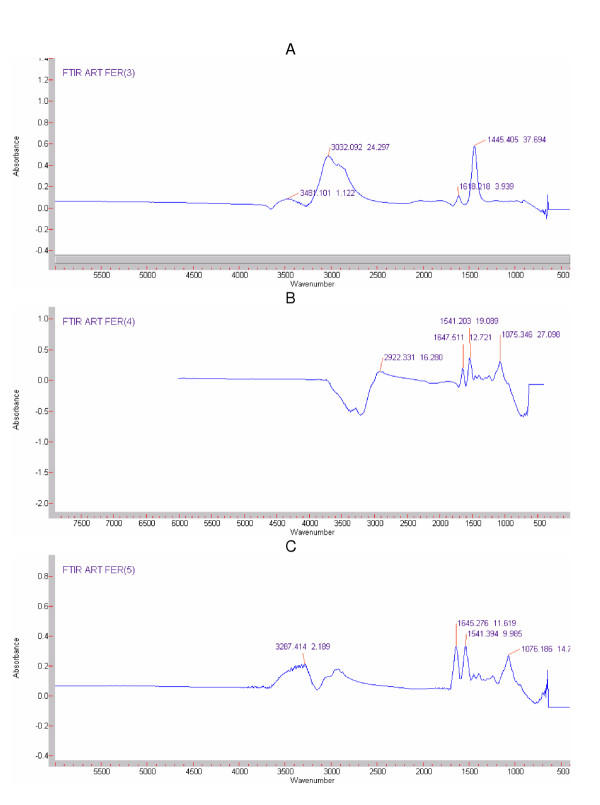
FTIR spectra of Fe_3_O_4 _magnetic nanoparticles (a) in nanopure water and Fe_3_O_4_-CHO (b), and pure CHO (c) prepared in phosphate buffer and then dissolved in nanopure water for FTIR analysis.

The binding of CHO to magnetic nanoparticles was confirmed by FTIR analysis. Figure 3 (a, b, and c) shows the FTIR spectra for "bare" Fe_3_O_4_, Fe_3_O_4_-CHO, and CHO in water, respectively. A characteristic band of NH_2 _was observed at 1618 cm^-1 ^in the "bare" Fe_3_O_4_nanoparticles. The NH_2 _group can be associated with NH stretch at 3400 cm^-1 ^which is not visible here, because of a possible hindrance by OH stretch from water. However, this band was not apparent in the spectra of Fe_3_O_4_-CHO suggesting that the binding of CHO to the nanoparticles involved this amino group and the carboxylic groups of CHO after being activated by Carbodiimide, as suggested by [4]. Peaks at 3032 cm^-1 ^and 1445 cm^-1 ^are more visible in Figure 3a (bare particles) and perceptible in Figure 3b (Fe_3_O_4_-CHO) and could be assigned to traces of residual ammonium hydroxide. The characteristic bands of proteins at 1647 and 1541 cm^-1, ^and 1645 and 1541 cm^-1^, in the spectra of Fe_3_O_4_-CHO, and CHO, respectively shows that cholesterol oxidase was effectively present in the samples, confirming the binding of cholesterol oxidase to Fe_3_O_4 _nanoparticles. The negative peak at 3400-2799 cm^-1 ^is possibly due to a reduced amount of water in the sample compared to the water used for background subtraction. The characteristic bands of proteins in the Fe_3_O_4_-CHO spectra were very weak compared to those in the spectra of cholesterol oxidase in water. The weakness of the peaks is due to the limited amount of CHO bound to the nanoparticles, in comparison to the amount dispersed in water.

### Cholesterol oxidase activity and binding kinetics

**Figure 4:**
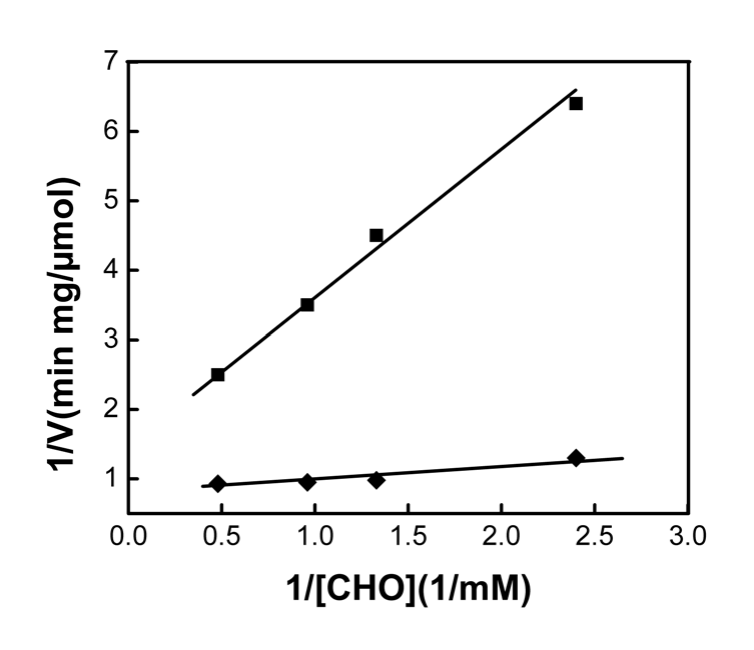
Lineweaver Burk plots of the initial rates of CHO (■) and (◆) Fe_3_O_4_-CHO at pH 7.4, from experimental data.

The kinetic parameters of the enzymatic reactions estimated by the Lineweaver-Burk plots of the initial rates of cholesterol oxidase from experimental data are presented in Figure 4. The Michaelis-Menten constants *V*_max _and *K*_m _for CHO were determined to be 0.67 μmol/min mg and 2.08 mM for the free enzyme and 1.64 μmol/min mg and 0.45 mM for the immobilized enzyme, respectively. The *V*_max_value of the bound CHO was 2.4 fold higher than that of the free, and the *K*_m _value of the bound CHO was 4.6 fold lower than that of the free CHO. The low *K*_m _reflects the high affinity to substrate [4]. The high affinity of the enzyme to the substrate may be explained by the fact that when binding onto the surface of the nanoparticles, the enzyme rearranged itself to present a better conformation. Since the secondary and tertiary structure of cholesterol oxidase play important roles in its activity [9], the rearrangement in structure and conformation may result in better availability of its active sites. The increase in affinity of the enzyme to the substrate upon binding to Fe_3_O_4 _nanoparticles contributed to an enhancement of the activity of the enzyme.

### Effect of pH

**Figure 5:**
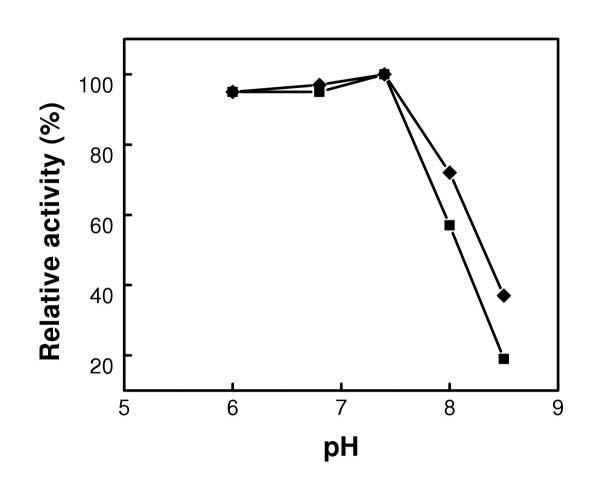
Effect of pH on the activities of free (■) and bound CHO (◆).

The effect of pH on the activities of the free and bound CHO was investigated in the pH range of 6–8.5 at 25°C and presented in Figure 5. In the pH range between 6 and 7.4 the activities of the free and bound CHO were quite similar and reached a maximum at pH 7.4. The activity then decreased from pH 8 to 8.5. In this range, the activity of the bound CHO was much higher than its free counterpart. This shows that the bound enzyme showed better tolerance to the variation of solution pH. The similarities in these activities in the pH range of 6 to 7.4 indicate that in these conditions, CHO did not suffer from any major activity constraint. Rather, this pH range appears to be suitable for CHO activity. It is well known that the ability of the amino acids at the active sites of the enzyme to interact with the substrate depends on their electrostatic state [16]. The decrease in activity observed at pH 8 and 8.5 shows that CHO faces some limitations as the pH increased toward more alkaline conditions. If the pH is not appropriate, the charge on one or all of the required amino acids is such that cholesterol can neither bind nor react properly to produce 4-cholesten-3-one.

### Thermal stability

**Figure 6:**
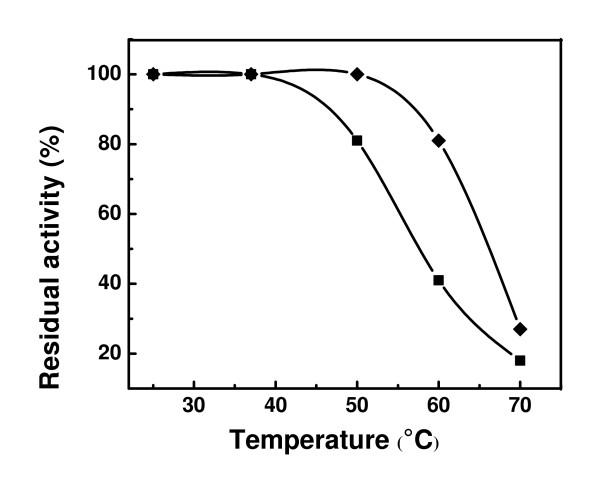
Thermal stability of free CHO (■) and Fe_3_O_4_-CHO (◆) at pH 7.4. The samples were stored at 50, 60, or 70°C for 40 min and the activities were then measured at 25°C.

The thermal stability of free and bound CHO was investigated after 40 min of storage in the temperature range of 25–70°C (Figure 6). There was no apparent change in activity in the free CHO as well as in the bound CHO, in the temperature range of 25–37°C. Above this temperature range, the residual activity decreased in both systems. However, the bound CHO showed higher retained activity than the free CHO. The remaining activity at 60°C was about 2 fold that of the free CHO. This proved that the thermal stability was significantly improved upon binding of CHO to magnetic nanoparticles. Table 1 shows the inactivation rates constants (*k*) at temperatures where the inactivation experiments were observed. The rate constants increased with increasing temperature and were higher for the free CHO than for bound CHO. As stated above, the binding to nanoparticles suggests a better resistance of the enzyme to temperature. We hypothesize that the bound enzyme could possibly undergo a conformational change and a spatial rearrangement that could slow down the folding process and denaturation of the enzyme.

**Table 1:** Inactivation rate constants (*k*) of the "bare" and bound CHO at various temperatures

Temperature (°C)	Free CHO	Fe_3_O_4_-CHO
	*k *(min^-1^)	*k *(min^-1^)

50	3.4 × 10^-2^	4.6 × 10^-3^
60	9.3 × 10^-2^	5.6 × 10^-2^
70	2.8 × 10^-1^	1.9 × 10^-2^

### Effect of temperature on enzyme activity and stability

**Figure 7:**
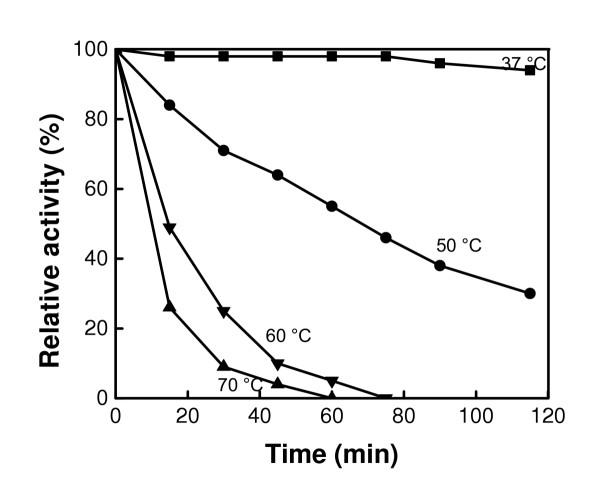
Effect of various temperatures on the activity Fe_3_O_4_-CHO at pH 7.4.

**Figure 8:**
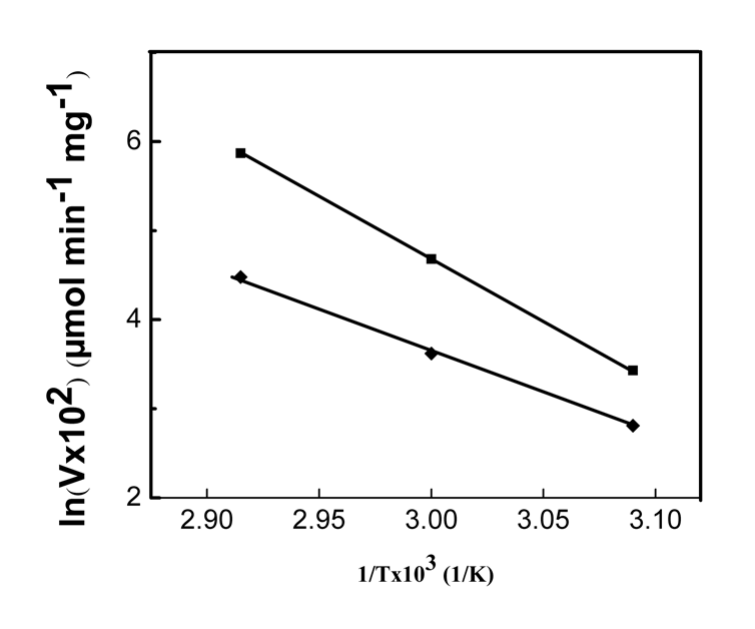
Arrhennius plots of the initial plots of the oxidation rates of cholesterol by free CHO (■) and Fe_3_O_4_-CHO (◆) for samples at 50, 60, or 70°C.

The effect of temperature on the activity of the free CHO was examined by measuring its relative activity when stored at various temperatures (Figure 7). It can be observed that at 37°C, the enzyme retained its activity for about 80 minutes before showing a slight decrease. At 50°C the activity decreased continuously to 35% after 110 min. A more severe decrease in activity occurred at 60 and 70°C, resulting in a complete loss of activity after 60 and 70 min, respectively. The decrease in activity may be attributed to a dramatic change in the structure of the enzyme that hindered the availability of the active sites, with a possible denaturation of the enzyme itself. The effect of temperature on the activities of free and bound CHO at pH 7.4 are displayed in the Arrhennius plots (Figure 8). Only temperatures (50, 60 and 70°C) at which perceptible changes in activity were observed were studied. The activation energies were calculated to be 13.6 and 9.3 KJ/mol for free and bound CHO, respectively. The low activation energy related to the bound CHO suggests that when bound to the magnetic nanoparticles, CHO seems to acquire a better orientation that reduces the energy barrier for activity.

### Storage stabilities

**Figure 9:**
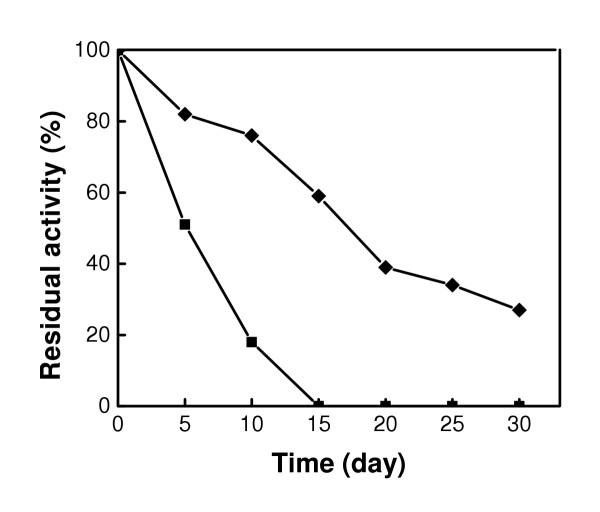
Storage stability of free CHO (■) and Fe_3_O_4_-CHO (◆). The activities measurements were performed at pH 7.4, at 25°C

The stability and activity of the enzyme are naturally reduced during storage. Figure 9 shows the storage stabilities of free and bound CHO at 25°C at pH 7.4. After 15 days, no residual activity was observed in free CHO. However, the residual activity of bound CHO was 59% during the same time period, and 27% after 30 days indicating a considerable enhancement on its stability. It has been argued that this higher stability of the bound enzyme was due to its fixation on the surface of magnetic nanoparticles, preventing the auto-digestion and thermal inactivity [3]. Another plausible explanation is that the binding of CHO on Fe_3_O_4 _nanoparticles might allow a better spatial orientation of the FAD prosphetic groups and the side chains of CHO providing a better stability to the enzyme.

## Materials and methods

### Materials

Cholesterol oxidase (EC 1.1.3.6), *Nocardia *sp. was purchased from VWR international (Pittsburgh, USA). Carbodiimide-HCl (1-ethyl-3-(3-dimethyl-aminopropyl), ammonium hydroxide reagent, Triton X-100, TRIS (Hydroxymethyl) aminomethane HCL, 4-cholesten-3-one, bovine serum albumin (BSA), iron (II) chloride tetrahydrate 97 %, and iron (III) chloride hexahydrate 99% were obtained from Sigma-Aldrich, St Louis (USA). The Biorad Protein Assay Dye Reagent Concentrate was purchased from Biorad Laboratories (Hercules, CA). Acetonitrile was obtained from EMD Chemicals, (New Jersey, USA).

### Preparation of magnetic nanoparticles

Magnetic nanoparticles (Fe_3_O_4_) were prepared by chemical co-precipitation of Fe^2+ ^and Fe^3+ ^ions in a solution of ammonium hydroxide followed by a treatment under hydrothermal conditions [4,5]. Iron (II) chloride and iron (III) chloride (1:2) were dissolved in nanopure water at the concentration of 0.25 M iron ions and chemically precipitated at room temperature (25°C) by adding NH_4_OH solution (30%), at a control pH (10–10.4). The suspensions were heated at 80°C for 35 min under continuous mixing and separated by centrifuging several times in water and then in ethanol at 2800 rpm. The purification step was used to remove impurities from Fe_3_O_4 _nanoparticles. The particles were finally dried in a vacuum oven at 70°C. The dried particles exhibited a strong magnetic attraction to a magnetic rod.

### Attachment of cholesterol oxidase onto magnetic nanoparticles

50–70 mg of magnetic nanoparticles was added to 1 mL of phosphate buffer (0.05 M. pH 7.4). The mixture was sonicated for 15 min after adding 0.5 mL of carbodiimide solution (0.02 g/mL in phosphate buffer (0.05 M. pH 7.4). Following the carbodiimide activation, 2 mL of cholesterol oxidase (0.25 mg/mL) was added and the reaction mixture was sonicated for 30 min at 4°C in a sonication bath and the mixture was centrifuged at 3000 rpm [17]. The precipitates containing Fe_3_O_4 _nanoparticles and Fe_3_O_4_bound cholesterol oxidase (Fe_3_O_4_-CHO) were washed with phosphate buffer pH 7.4 and 0.1 M Tris, pH 8.0, 0.1 M NaCl and then used for activity and stability measurements. NaCl was added to enhance the separation of the magnetic nanoparticles [3].

### Determination of immobilization efficiency

The amount of protein in the supernatant was determined by a colorimetric method at 595 nm using the Biorad Protein Assay Reagent Concentrate with bovine serum albumin (BSA) as the protein standard. The amount of bound enzyme was calculated from:

*A *= (*C*_*i *_- *C*_*s*_)*V   (2)

Where *A *is the amount of bound enzyme, *Ci *and *Cs *is the concentration of the enzyme initially added for attachment, and in the supernatant, respectively (mg-mL^-1^), *V *is the volume of the reaction medium (mL).

### Characterization

The size of Fe_3_O_4 _nanoparticles and Fe_3_O_4_-CHO was characterized by transmission electron microscopy (TEM, JEM 1200 EXII, JEOL USA) and structure by Fourier Transform Infrared (FTIR) spectroscopy (Biorad FTS 6000, Cambridge, MA). The samples for TEM analysis were prepared by placing a drop of the magnetic nanoparticles dispersed in nanopure water onto a copper grid and evaporated in air at room temperature. Before preparing a sample onto the copper grid, the dispersed solution was sonicated for 4 min to obtain better particle dispersion. The binding of CHO onto the magnetic nanoparticles was investigated using FTIR. CHO and Fe_3_O_4_-CHO samples in phosphate buffer and Fe_3_O_4 _particles were dissolved in nanopure water for FTIR analysis.

### Activity measurement

The activity of bound CHO was determined by measuring the initial oxidation rates of cholesterol by cholesterol oxidase at given temperature following the increase of 4-cholesten-3-one concentration at 240 nm, using a Beckman Du Spectrometer. A solution of cholesterol was prepared by dissolving 4.8 g of cholesterol in 10 mL of 2-propanol. A phosphate buffer solution (0.05 M. pH 7.4) containing 4% of Triton-100 was added to the mixture to result in a 0.26 M cholesterol solution. The mixture was gently heated until the solution was clear. To start the enzymatic reaction, 5 ml of cholesterol solution was added to 15 mL centrifuge test tubes containing Fe_3_O_4_-CHO, and mixed by vortex. A solution of free CHO of the same concentration was used to evaluate the activity of the free enzyme. The solution was incubated at various temperatures (25–70°C) at specific intervals of time (1 h) and centrifuged at 3000 rpm for 5 min to separate the supernatant from Fe_3_O_4_-CHO. 10 μL aliquots of the supernatant were then taken and the concentration of 4-cholesten-3-one was assessed. Before measuring the amount of 4-cholesten-3-one in a sample, the activity of the free enzyme was stopped by adding an equal volume of acetonitrile to the reacting solution [18]. Each kinetic measurement was the average of duplicate replications.

### Thermal stability of free and immobilized enzyme

The thermal stability of free and Fe_3_O_4_-CHO were determined by measuring the residual activity of the enzyme at 25°C, after being exposed to different temperatures (25–70°C) in phosphate buffer (0.05 M, pH 7.4) for 40 min. Aliquots of the reacting solution were taken at time intervals (every 30 min for 7 hours) and assayed for enzymatic activity as described above. The first order inactivation rate constant, *k *was calculated from the equation:

In *A *= In *A*_0 _- *kt*   (3)

where *A*_*0 *_is the initial activity, *A *is the activity after a time t (min), *k *is the reaction constant.

### Effect of temperature on enzyme activity

The effect of temperature on the free CHO and Fe_3_O_4_-CHO was estimated by determining the concentration of 4-cholesten-3-one in samples at various temperatures. A solution of cholesterol was added to the various centrifuge test tubes containing bound or free enzymes. The test tubes were stored in a water bath at specific temperatures (25, 37, 50, 60, and 70°C). At time intervals, the concentration of 4-cholesten-3-one was determined by spectrophotometric analysis.

### Storage activity

The storage stability was evaluated by determining the concentration of 4-cholest-en-3-one at room temperature at time intervals (5 days). Test tubes containing Fe_3_O_4_-CHO or free enzyme solution were stored at 25°C in phosphate buffer (0.05 M. pH 7.4) for 30 days. Thereafter, 5 mL of cholesterol was added. The storage stability of the free and bound cholesterol oxidase was determined by assaying for their residual activity.

### Determination of kinetics parameters

The kinetic parameters of free CHO and Fe_3_O_4_-CHO, *K*_m_ and *V*_max_ were determined by measuring initial rates of oxidation of cholesterol (1.3–5.2 mM) by CHO (0.25 mg/mL) in phosphate buffer pH 7.4 at 25°C.

## Conclusions

Magnetic nanoparticles were synthesized by thermal co-precipitation of ferric and ferrous chlorides. The binding of CHO to the particles was confirmed by FTIR spectroscopy and the size characterized by TEM. The binding efficiency was between 98 and 100% irrespective of the amount of particles used. Kinetic studies of the free and bound CHO revealed that the stability and activity of CHO were significantly improved upon binding to nanoparticles. Furthermore, the bound enzyme exhibited a better tolerance to pH, temperature and substrate concentration. The activation energy indicated that the binding of CHO onto Fe_3_O_4 _magnetic nanoparticles reduced the energy barrier for CHO activity. As a result of the binding to the magnetic nanoparticles, the storage stability of CHO was considerably enhanced. This higher stability of the Fe_3_O_4_-CHO is attributable to its possible fixation on the surface of the particles preventing auto-digestion and thermal inactivity. In addition, the binding on Fe_3_O_4 _nanoparticles might allow a better spatial orientation of the FAD prosphetic groups and the side chains of CHO to provide better stability to the enzyme. The overall improvements observed in activity, stability, and functionality of CHO resulted from structural and conformational changes of the bound cholesterol oxidase. The study may be useful in improving the stability and activity of cholesterol oxidase, and will contribute to more efficient use of this enzyme.

## List of Abbreviations used

CHO: Cholesterol oxidase

TEM: Transmission electron microscopy

FTIR: Fourier Transform Infrared

BSA: Bovine serum albumin

## Authors' contributions

Drs Gilles K Kouassi and Joseph Irudayaraj were the primary authors. They were responsible for the concept, experimental plan, and analysis. Dr Gregory McCarty was the secondary author and contributed to the overall effort.
